# Unique Clones of *Vibrio cholerae* O1 El Tor with Haitian Type *ctxB* Allele Implicated in the Recent Cholera Epidemics from Nigeria, Africa

**DOI:** 10.1371/journal.pone.0159794

**Published:** 2016-08-01

**Authors:** Akinsinde Kehinde Adewale, Gururaja Perumal Pazhani, Iwalokun Bamidele Abiodun, Oluwadun Afolabi, Olukoya Daniel Kolawole, Asish K. Mukhopadhyay, Thanadarayan Ramamurthy

**Affiliations:** 1 Clinical Science Division, Nigerian Institute of Medical Research, Lagos, Nigeria; 2 Division of Molecular Microbiology, National Institute of Cholera and Enteric Diseases, Kolkata, India; 3 Molecular biology & Biotechnology Division, Nigerian Institute of Medical Research, Lagos, Nigeria; 4 Olabisi Onabanjo University, Sagamu, Ogun, Nigeria; 5 Danifol foundation, Onipanu, Lagos, Nigeria; The Australian National University, AUSTRALIA

## Abstract

**Background and Objectives:**

The antimicrobial susceptibility patterns and genetic characteristics of *Vibrio cholerae* O1, which is responsible for several cholera epidemics in Nigeria, are not reported in detail since 2007. In this study, we screened *V*. *cholerae* O1 El Tor biotype isolates from cholera cases and water samples from different states to investigate their phenotypic and genetic attributes with special reference to their clonality.

**Results:**

All the *V*. *cholerae* O1 biotype El Tor isolates isolated during 2007–2013 were susceptible to fluoroquinolones and tetracycline, the drugs currently used in the treatment of cholera cases in Nigeria. Emergence of CT genotype 7 (Haitian type of *ctxB* allele) was predominantly seen among Ogawa serotype and the CT genotype 1 (classical *ctxB* allele) was mostly found in Inaba serotype. Overall, *V*. *cholerae* O1 from clinical and water samples were found to be closely related as determined by the pulsed-field gel electrophoresis. *V*. *cholerae* isolates from Abia, Kano and Bauchi were found to be genetically distinct from the other states of Nigeria.

**Conclusion:**

Fecal contamination of the water sources may be the possible source of the cholera infection. Combined prevalence of Haitian and classical *ctxB* alleles were detected in Ogawa and Inaba serotypes, respectively. This study further demonstrated that *V*. *cholerae* O1 with the *ctxB* has been emerged similar to the isolates reported in Haiti. Our findings suggest that the use of fluoroquinolones or tetracycline/doxycycline may help in the effective management of acute cholera in the affected Nigerian states. In addition, strengthening the existing surveillance in the hospitals of all the states and supply of clean drinking water may control cholera outbreaks in the future.

## Introduction

Despite several efforts, cholera continues to occur as a major public health problem in African countries with high rates of morbidity and mortality. In 2013 alone, 22 countries from the African continent have reported 56,329 cholera cases, including 1366 deaths with case fatality rate (CFR) of 2.43% [1]. Appearance of epidemic cholera caused by toxigenic *Vibrio cholerae* was first reported in Nigeria during 1971 and since then, this devastating disease became endemic in this country [2, 3]. Several outbreaks of cholera have been reported with frequent occurrence of *V*. *cholerae* O1 biotype El Tor, serotype Ogawa [3]. During outbreaks, prevalence of culture confirmed cases of cholera in Nigeria vary from 10 to about 50% [3, 4]. Due to the initiation of surveillance and effective management, the CFR of cholera has been decreased in Nigeria from 15% in 1995/96 to 5% in 1997 and 2% in 1999 [5]. However, the intermittent large cholera outbreaks such as the one observed in 2010 with 41,787 cases and 1,716 deaths has increased the CFR to 4.1% [6].

The phenotypic changes of *V*. *cholerae* O1 are very frequent in Africa. Earlier studies conducted in Zaria during 1975–1986 showed the prevalence of Hikojima serotype during 1976–1978, but Ogawa became dominant from 1984 to 1986 [7]. During 1995–1999, *V*. *cholerae* O1 Inaba was major serotype in Nigeria [5], which was replaced again by Ogawa in the following years. In 2010, more than 40,000 cases of cholera have been reported with 780 deaths in Nigeria along with the emergence of multidrug resistant atypical El Tor *V*. *cholerae* O1 Ogawa [3, 8, 9]. Compared to previous years, the incidence of cholera was less in Nigeria during 2012 with 585 cases including 13 deaths with the CFR of 3.0% [1]. Since most parts of Nigeria depend on either well or stored rain water, the water-borne infections are high in this country, predominantly with typhoid, bacillary dysentery and cholera [10]. The isolation rate of *V*. *cholerae* O1 sometimes go up to 18% in stored rainwater and 23.6% of well water samples [10]. The seventh cholera pandemic is caused by the robust El Tor biotype compared to the less adopted but more toxigenic classical biotype of *V*. *cholerae* O1. Based on multiple genetic mutations in the gene (*ctxB*) encoding the B-subunit of the cholera toxin, several investigations show the emergence of atypical El Tor biotype strains that are highly pathogenic [11]. The genetic mutations associated with this strain result in the replacement of histidine by asparagine at position 20 (H20N), tyrosine replacement by histidine at position 39 (Y39H) and isoleucine replacement by threonine at position 68 (I68T) of the CtxB peptide subunit [12]. This study was undertaken to detect the phenotypic and genetic changes of *V*. *cholerae* O1 isolated during 2007–2013 from cholera cases and borehole, well, stream and tap water sources in different states of Nigeria.

## Materials and Methods

### Study design

This was a mixed-method cross-sectional study on cholera epidemics that occurred in nine states in Nigeria during 2007–2013. At different time points of this survey, patients suspected to have cholera during 2009–2011 and 2013 were enrolled into the study. The patients with symptoms of cholera episodes such as watery diarrhea, vomiting, dehydration, lethargy etc., were hospitalized at the designated cholera treatment camps. The affected states were Bauchi, Borno and Gombe in the North-east, Kano in north-west, Ilorin (Kwara state) in north-central; Abia (south-east), Lagos, Ogun and Osun states (south-west). In this study, cholera patients hospitalized at the designated cholera treatment camps were enrolled. Before administration of antibiotics, fecal samples or rectal swabs of hospitalized patients who consented to participate at the time of treatment were collected into sterile leak-proof tubes or Cary Blair transportation medium (Difco, Sparks, MD, USA). Samples were taken to the designated cholera surveillance laboratory of each state for the analysis. In order to identify the source of the infection, geographic information system was used to map water sources (a minimum of 5 Km apart) in the communities surrounding each of the treatment camps. Randomized sampling of water from river/stream, ponds and wells has been made from cholera epidemic communities in the affected nine states. In addition, *V*. *cholerae* O1 isolates from stools and well water during 2007–2008 cholera epidemics in Kano, Nigeria were obtained from Central Public Health Laboratory (CPHL), which is a national repository laboratory for cholera control program under Federal Ministry of Health. These retrospective isolates were also used in the clonal analysis along with those isolated during 2009–2013 from clinical samples. The CPHL is a repository for *V*. *cholerae* isolates recovered during epidemics in different states of the country, but not involved directly with cholera cases. However, members of the CPHL participate in infectious disease surveillance activities in the country when called upon by the CPHL. Patients with other associated illness or used antibiotics before hospitalization were not included in this study. Based on the dehydration status, patients were treated with oral rehydration salts/intravenous fluids and electrolytes.

### Ethical Statement

The prospective study conducted during 2009–2013 cholera epidemics and the retrospective isolates collected during 2007 were approved by the Ethical committee of Olabisi Onabanjo University. Approvals of this study were sanctioned by the State Ministry of Health of the affected states and also from CPHL management and Kano State Ministry of Health. The samples were collected specifically for this study. Letter of collaboration/support was also obtained from the State Ministry of Health of the affected states to have access to the designated treatment camps for sampling. Written informed consent was obtained from each patient before enrolment. The consent of each child participant was obtained through the parent or guardian. *V*. *cholerae* O1 collected from stool and well water samples during the 2007 cholera epidemics in Kano state and stored as archive isolates in the culture repository of CPHL was used for clonal analysis after obtaining approvals from the CPHL management and Kano State Ministry of Health. This study was conducted in line with the declaration of Helsinki on safety, confidentiality and beneficence of human subjects. Both the study data and samples were anonymized.

### Bacterial isolates

Stool/rectal swabs were collected from cholera patients who received treatment in designated medical camps. Water samples were collected from cholera epidemic communities in the affected nine states. Samples were immediately processed according to WHO standard bacteriological procedures for *V*. *cholerae* at the designated Government laboratories (http://www.who.int/csr/resources/publications/drugresist/VIAMRManual.pdf). Suspected *V*. *cholerae* colonies that showed typical morphology on thiosulphate-citrate bile salt-sucrose (TCBS) were Gram stained and serotyped using specific antisera (Bio-Rad, Hercules, CA, USA). A total of 122 isolates of *V*. *cholerae* were sent to National Institute of Cholera and Enteric Diseases, Kolkata, India for further confirmation and detailed molecular characterization. *Escherichia coli* strain ATCC 25922 was used as the quality control strain in the antimicrobial susceptibility testing. In the pulsed-field gel electrophoresis (PFGE), *Salmonella enterica* serotype Braenderup strain H9812 was sued as the molecular size standard.

### Antimicrobial susceptibility testing

Antimicrobial susceptibility testing was performed using the disc diffusion method in accordance with Clinical and Laboratory Standards Institute [13] using commercially available discs (BD, Sparks, MD, USA). These discs include ampicillin (AM) (10 μg), azithromycin (AZM) (15 μg), ceftriaxone (CRO) (30 μg), chloramphenicol (C) (30 μg), ciprofloxacin (CIP) (5 μg), trimethoprim/sulfamethoxazole (SXT) (1.25μg/23.75μg), nalidixic acid (NA) (30 μg), norfloxacin (NOR) (10 μg), ofloxacin (OFX) (5 μg), streptomycin (S) (10 μg) and tetracycline (TE) (30 μg).

### Preparation of template DNA for PCR

All the test isolates were grown overnight at 37°C in Luria Bretani broth (Difco) and about 400 μl of culture was transferred to microfuge tube followed by centrifugation at 8000 rpm for 5 min. The culture supernatant was aspirated and the cell pellet was resuspended to the original volume with nuclease free water. About 200 μl of the cell suspension was used for extraction of the total nucleic acid using an automated system (NucliSens EasyMAG; bioMe´rieux, Marcy l'Etoile, France). After quantification, the diluted DNA was used in the PCR assays.

### PCR amplification for the detection of virulence genes and typing of *ctxB* alleles

The presence of cholera toxin (CT) A-subunit gene (*ctxA*) and *tcpA* variants (the major structural subunit gene of the toxin-coregulated pilus) of classical and El Tor biotypes were determined by a multiplex PCR assay [14]. A double-mismatch-amplification mutation assay PCR (DMAMA-PCR) was used to identify the *ctxB* alleles of classical, El Tor, and Haitian types [15]. From the positive isolates of DMAMA-PCR, randomly selected isolates were confirmed by PCR-sequencing.

### Pulsed-field gel electrophoresis (PFGE)

PFGE analysis of *NotI*-digested genomic DNA from representative isolates was performed using a CHEF Mapper System (Bio-Rad) according to the PulseNet standardized PFGE protocol for subtyping of *V*. *cholerae* [16]. The analysis of the PFGE profiles of *V*. *cholerae* isolates were compared using BioNumerics software (Applied Maths,sint-Martens-Latem, Belgium). The similarities between the isolates were evaluated using the Dice coefficient method. Cluster analysis was carried out using the unweighted-pair group method using average linkages (UPGMA).

### *ctxB* region analysis by sequencing

The isolates which yielded negative bands for the Haitian *ctxB* allele by DMAMA-PCR, were selected for DNA sequencing. The whole *ctxB* gene was amplified by PCR and the amplicons were purified using a QIAquick Gel extraction kit (QIAGEN GmbH, Hilden, Germany). Sequencing reactions were set using a Big Dye Terminator Cycle Sequencing Ready Reaction Kit (Applied Biosystems, Foster City, CA, USA). Nucleotide sequencing was performed in an ABI Prism 3200 Automatic Sequencer (Applied Biosystems). To analyze the identity of the sequences, comparisons were made using the BLAST program (http://www.ncbi.nlm.nih.gov/blast).

## Results

Of the 122 isolates tested, 115 were confirmed as *V*. *cholerae* O1 and the rest 7 as *Aeromonas* spp. The *Aeromonas* isolates were initially identified as *V*. *cholerae* non-O1, non-O139 based on the sucrose fermentation in TCBS (yellow color colonies) and non-agglutination with O1/O139 antisera. A total of 115 isolates consisting of 92 from clinical and 23 from water sources were further analyzed. The details of *V*. *cholerae* O1 isolated from water samples collected in different states of Nigeria and their sources are shown in Table 1.

**Table 1:** Distribution of *V*. *cholerae* O1 in water samples of Nigerian states.

State	Source of water samples				Total
	Borehole	Well	Stream	Tap	
Abia	1	1	2		4
Bauchi		2	1		3
Borno		1	2		3
Gombe		1	1		2
Ilorin			1		1
Kano			2		2
Lagos		1		1	2
Ogun		1	1	1	3
Osun	1	1	1		3
**Total**	**2**	**8**	**11**	**2**	**23**

Results of antimicrobial susceptibility test showed diversity in the resistance profiles. All the tested isolates were susceptible to ampicillin, azithromycin, ceftriaxone, ciprofloxacin, chloramphenicol, norfloxacin, ofloxacin tetracycline and doxycycline. Details of *V*. *cholerae* isolated from different states and their resistance profile in the form of antibiogram are presented in Table 2. Interestingly, *V*. *cholerae* O1 Inaba isolates from both clinical as well as water samples were susceptible to nalidixic acid. *V*. *cholerae* isolates from Borno, Gombe and Lagos were found to be fully resistant to nalidixic acid, streptomycin and trimethoprim/sulfamethoxazole (Table 2).

**Table 2:** Location, source, serotype, cholera toxin (*ctxB*) allele and resistant profiles of the *V*. *cholerae* O1 El Tor biotype isolates.

State/Location	Source		O1 Serotype		*ctxB* allele*		Resistant profile (%)
	Clinical	Water	Ogawa	Inaba	Classical	Haitian	
Borno	17	3	20	0	0	7	NA S SXT (100)
Bauchi	7	3	5	5	5	3	S SXT (50); NA S SXT (50)
Abia	16	4	20	0	0	8	NA S SXT (95); NA SXT (5)
IIorin	3	1	4	0	0	2	NA S SXT (75); NA S (25)
Kano	5	2	0	7	6	0	S (57); S SXT (29); SXT (14)
Gombe	7	2	9	0	0	4	NA S SXT (100)
Ogun	21	3	24	0	0	6	NA S SXT (88); NA S (8); S SXT (4)
Osun	11	3	14	0	0	4	NA S SXT (93); NA S (7)
Lagos	5	2	7	0	0	3	NA S SXT (100)
**Total**	**92**	**23**	**103**	**12**	**11**	**37**	

Abbreviations: NA, nalidixic acid; S, streptomycin and SXT, trimethoprim/sulfamethazole. Number in parenthesis represents percentage resistance type.

*atypical El Tor (ctx*B* allele, genotype 7) and classical El Tor (*ctxB* allele, genotype 1) analyzed by PFGE.

All the tested isolates harbored *ctxA* and *tcpA* (El Tor) genes. DMAMA-PCR assay results are shown in the Table 2. One hundred and three (89.6%) isolates were identified to have the Haitian *ctxB* allele. However, nine isolates were negative in the DMAMA-PCR assay. When sequenced full length of the *ctxB* gene, we found that 5 and 4 isolates harbored Haitian and classical type alleles of *ctxB*, respectively. Classical *ctxB* allele was identified in 11 out of 12 Inaba isolates from Bauchi and Kano regions (Table 2).

**Fig 1:**
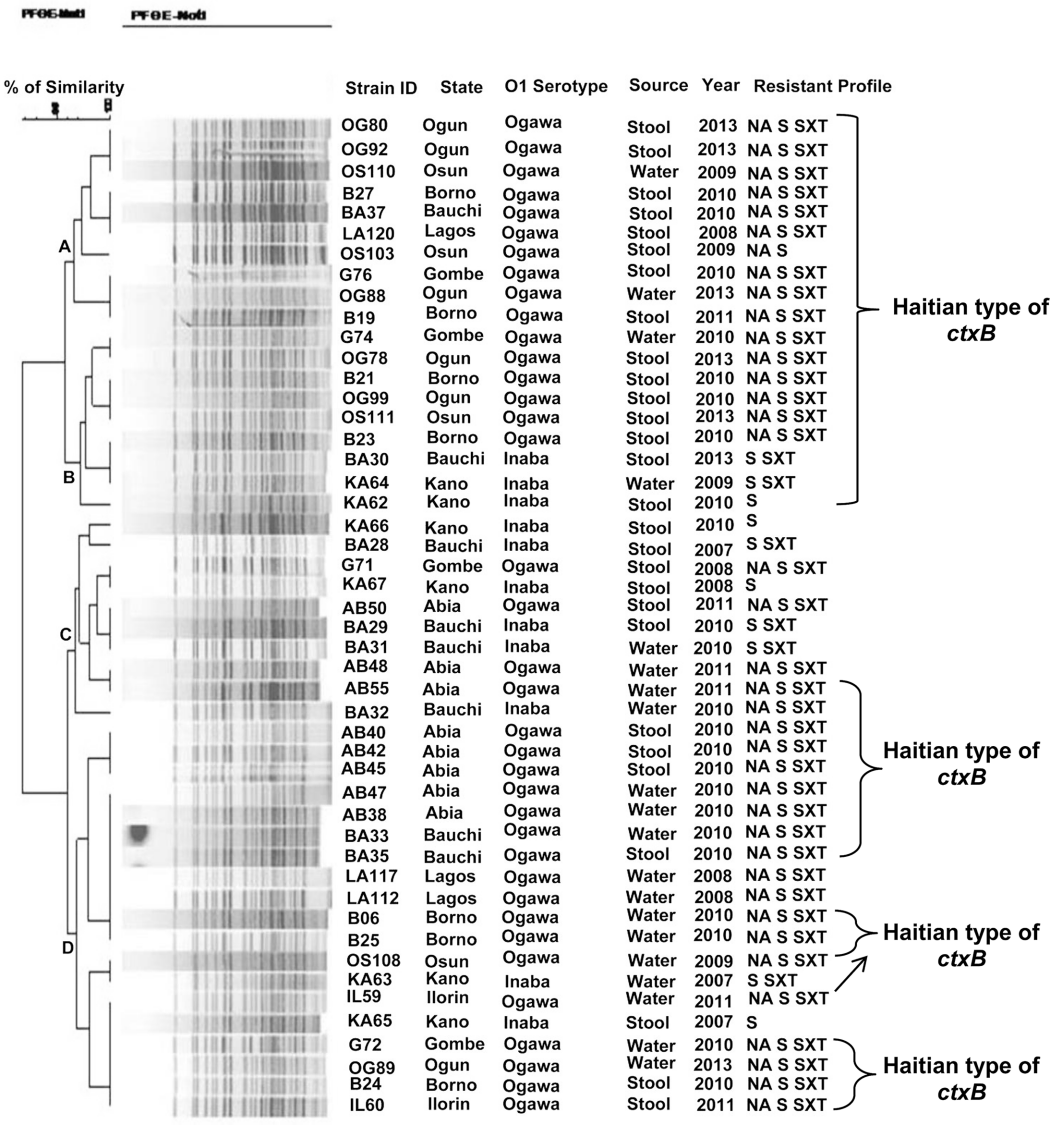
PFGE Profiling of *V*. *cholerae* O1. Dendogram of *Not1* digested PFGE patterns of *V*. *cholerae* O1 El Tor biotype. PFGE analysis revealed that all the isolates shared 95 to 100% similarity in *Not1* digested DNA pattern. Although they were isolated from different geographical regions of Nigeria in different years, they were found clonal and had different antimicrobial resistance patterns.

Clonal lineage of *V*. *cholerae* isolated from diverse sources and states were compared in the PFGE analysis. In this analysis, all the isolates shared 95 to 100% similarity in *Not*I digested DNA pattern (Fig 1). In the dendrogram, water and clinical isolates from different states of Nigeria were placed in clades A to D (Fig 1). However, majority of the *V*. *cholerae* isolated from water samples were placed in clade D. These isolates displayed identical PFGE pattern that matched with clinical isolates from the same and neighboring states. Isolates detected with Haitian *ctxB* allele were placed in clades A and B. Clades C and D had both the Haitian and classical types of *ctxB* (Fig 1). Although the *V*. *cholerae* O1 were isolated from different geographical regions of Nigeria for many years. They were found to be clonal with different antimicrobial susceptibility patterns.

## Discussion

Based on the amino acid positioning in the B-subunit of the CT encoding gene (*ctxB*) of *V*. *cholerae*, several CT genotypes have been reported [11]. The classical vibrios have CT genotype 1 (classical *ctxB* allele), with a difference of only single nucleotide (cytosine instead of adenine) at position 83. The subsequently appeared El Tor vibrios were reported to have many CT genotypes. *V*. *cholerae* O1 isolates from Haitian cholera outbreak had unique mutation in the 58^th^ amino acid of CtxB and hence known as Haitian *ctxB* allele or CT genotype 7. Spread of *V*. *cholerae* harboring this allele is now widespread in many Asian countries associated with cholera outbreaks [11].

In this study, 103 out of the 115 epidemic isolates of *V*. *cholerae* O1 were identified during the 2007–2013 cholera outbreaks in Nigeria. Majority of them carried the atypical El Tor *ctxB-*7 allele, which is also known as CT genotype 7. This study has further confirmed the involvement of atypical El Tor *V*. *cholerae* in cholera outbreaks in Nigeria as recently reported in a comprehensive study by Marin *et al* [9]. Of interest is the fact that a less expensive technique like DMAMA compared to whole genome sequencing used by Marin *et al* [9] was employed in the present study to identify the atypical El Tor strains. Using DMAMA, we have been able to show that the atypical El Tor *V*. *cholerae* has been causing cholera outbreaks in Nigeria since 2007 and has spread to various states in the country. The CT genotype 7 alleles, which defined atypical El Tor identification is also similar to the Haitian *ctxB*, making atypical El Tor *V*. *cholerae* a global cholera problem. Previously, Marin *et al*. [9] reported Haitian *ctxB* among *V*. *cholerae* O1 isolated during the 2009–2010 outbreaks in Maiduguri and Bauchi, North Eastern Nigeria as well as Ile-Ife in the south-Western region. *V*. *cholerae* from these northern states and Ile-Ife in Osun state were also among those identified to have Haitian *ctxB* allele.

The identification of the highly virulent atypical El Tor *V*. *cholera*e since 2007 in this study may explain the recurrence of cholera epidemics with alarming case fatality rates in the country in the last 10 years. Based on our findings, we can also speculate that the currently predominating Haitian *ctxB* allele carrying epidemic *V*. *cholerae* O1 might have its origin from Lagos before spreading to other states of the country. This also suggests the existence of *V*. *cholerae* similar to the one found in Haiti about 6 years before it was reported [9]. Apart from Lagos, the isolates having Haitian *ctxB* allele were also detected from Osun in 2009, four years earlier than their detection in 2013 in Ile-Ife, another settlement in Osun state. Detection of *V*. *cholerae* with Haitian *ctxB* allele in Bauchi, Abia and Ilorin further provides evidence for its wide spread in Nigeria. The Haitian *ctxB* carrying *V*. *cholerae* O1 is reportedly has its origin from East Asia and increasingly reported in Africa and other countries [11, 15]. Cholera endemic African countries where the atypical El Tor *V*. *cholerae* O1 has been reported include Mozambique [17], Ghana [18], Angola [19], Zambia [20], Zimbabwe [21] and Kenya [22].

Another interesting finding from this study is that all the Haitian type of *ctxB* allele carrying isolates belongs to the Ogawa serotype. Except in one isolate (BA32), the classical *ctxB* allele was detected in Inaba serotype. This serotype dependent carriage of the Haitian *ctxB* was not documented in any of the previous studies [9, 15, 23]. Mercy *et al*. [22] reported that presence of classical allele of *ctxB* was restricted to the Inaba serotype of *V*. *cholerae* O1 and absence of Haitian *ctxB* allele among epidemic isolates detected during 2007–2010 cholera outbreaks in Kenya [22].

Apart from being more pathogenic compared to the classical El Tor *ctxB* allele carriers, the isolates with Haitian *ctxB* allele also displaced multidrug resistance phenotype [9]. This property was also confirmed in the present study with 94% isolates that showed resistance to trimethoprim/sulphamethozaxole, streptomycin and nalidixic acid. The antimicrobial susceptibility testing results in the present study does not support the use of nalidixic acid, co-trimoxazole and streptomycin in the clinical management of cholera. This is also in agreement with the other recent findings from Nigeria [4, 9]. In addition, *V*. *cholerae* O1 resistance to nalidixic acid, trimethoprim/sulphamethazole and streptomycin have been well documented in other cholera endemic countries [23–25].

In this study, the *V*. *cholerae* isolates were found to be generally susceptible to doxycycline, tetracycline and fluoroquinolones, which are used as first and second-line antibiotics in the treatment of cholera cases in Nigeria. Hence, our findings suggest that the treatment of cholera patients with doxycycline and tetracycline in Nigeria may be continued. However, recent studies in Nigeria and other cholera endemic countries suggest cautionary use of fluoroquinolones as second line antibiotics [20, 26]. It is important to note that reduced susceptibility to fluoroquinolones was reported in Nigeria among *V*. *cholerae* isolated during 2009–2010 epidemics in Maiduguri, Bauchi and Ile-Ife, in North Eastern and South-Western Nigeria [9].

Analysis of the *Not*1 PFGE profiles of *V*. *cholerae* revealed 4 different clusters among Nigerian isolates. All the tested *V*. *cholerae* O1 isolates had a similarity matrix of >95%. Based on this analysis, there were no remarkable genetic differences between clinical and water sample isolates. This suggests contamination of the water sources by this pathogen and thus may act as a reservoir in the transmission of disease. In the PFGE analysis, we found that clonal relations of classical *ctxB* carrying Inaba/Ogawa isolates and Haitian *ctxB* carrying isolates were the same. These isolates were clustered in clades C and D. Similar observation on genetic lineage was reported in Nigeria [9] and Kenya [22]. As reported previously in El Tor vibrios, the Haitian type isolates encountered in this study showed extensive clonal diversity in the PFGE [9]. Beyond mutation within the *ctxB*, we assume that there may be changes in other genes so as to help the organisms for better survival under unfavorable environmental conditions.

Detection of Haitian type *ctxB* allele in *V*. *cholerae* has influenced the public health officials to examine and monitor the progression of new clones if any in the country. This activity can be further strengthened with an active surveillance system for systematic monitoring of cholera infection. Provision of potable water supply should be in place, especially in communities, which are mainly depending on stream water. Our study clearly indicated presence of *V*. *cholerae* O1 in drinking water sources that may be due to fecal contamination. The health care providers should be properly trained to use the rapid diagnostic tests for early detection of cholera so as to manage patients in situations like cholera outbreaks in this country.

## Abbreviations

CT: cholera toxin
CRF: case fatality rate
CPHL: Central Public Health Laboratory
DMAMA: double-mismatch-amplification mutation assay
PFGE: pulsed-field gel electrophoresis
